# Dicrocoeliosis in extensive sheep farms: a survey

**DOI:** 10.1186/s13071-019-3609-2

**Published:** 2019-07-12

**Authors:** Antonio Scala, Claudia Tamponi, Giorgia Dessì, Giampietro Sedda, Giuliana Sanna, Silvia Carta, Andrea Corda, Philippe Jacquiet, Antonio Varcasia, Ciriaco Ligios

**Affiliations:** 1 Laboratory of Parasitology, Veterinary Teaching Hospital, Department of Veterinary Medicine, University of Sassari, Sassari, Italy; 2 Inter-University Center for Research in Parasitology (CIRPAR), Via della Veterinaria 1, 80137 Napoli, Italy; 3 UMT Santé des Troupeaux de Petits Ruminants, Ecole Nationale Vétérinaire de Toulouse, Toulouse, France; 4 Istituto Zooprofilattico Sperimentale della Sardegna G. Pegreffi, Via Duca degli Abruzzi 8, Sassari, Sardinia Italy

**Keywords:** Sheep, Breeding, Trematoda, *Dicrocoelium dendriticum*, Epidemiology

## Abstract

**Background:**

This study investigated the epidemiological and molecular aspects of dicrocoeliosis in extensive sheep farms.

**Methods:**

From 2013 to 2014, copromicroscopical analyses in 190 dairy sheep farms and anatomo-pathological inspections in six slaughterhouses were carried in Sardinia, Italy. Rectal faecal samples were analyzed using the FLOTAC® method, and anatomo-pathological examinations were based on detecting thickened terminal bile ducts (TTBDs). In addition, genetic analyses were conducted on representative DNA samples of adult *Dicrocoelium* spp.

**Results:**

Ninety-seven (51.1%) out of 190 sheep farms were coprologically positive for *Dicrocoelium* spp. In the liver, on the surface and cut surface, TTBDs were reported in 40.1% (309/770) and 15.3% (118/770) of the animals examined, respectively, with an overall prevalence of 25.5% (196/770). No intraspecific genetic variation was observed among the *Dicrocoelium dendriticum* isolates.

**Conclusions:**

Our survey reveals the widespread presence of *D. dendriticum* in Sardinia, although seasonal, geographical and climatic conditions might be key factors in modulating the infection prevalence. Examining typical lesions due to *D. dendriticum* in the liver in abattoirs can be used as a marker for tracking chronic dicrocoeliosis infection.

## Background

Dicrocoeliosis is a disease caused by several species of the genus *Dicrocoelium* Dujardin, 1845 (Trematoda: Digenea), which live in the hepatic bile ducts and gall-bladder of domestic and wild ruminants [1]. Liver lesions due to dicrocoeliosis, such as abscesses, granulomas and fibrosis, as well as bile duct proliferation have also been described in the New World camelids (llamas and alpacas) [2–4]. Occasionally, *Dicrocoelium* spp. can also infect rabbits, pigs, dogs, horses and humans [5]. The various species of *Dicrocoelium* have different geographical distributions, with *D. dendriticum* being the most widespread globally, being found in Europe, Asia (China and the Indo-Malayan region), Japan, North Africa and Australia [3, 5], while *Dicrocoelium hospes*, *Dicrocoelium chinensis* and *Dicrocoelium suppereri* [3] have a limited distribution in Africa, Asia and some areas of western Europe, respectively [6–9]. To complete its life-cycle, *Dicrocoelium* develops within the body of some land snails and ant species, which act as first and second intermediate hosts, respectively [10].

Dicrocoeliosis is commonly considered to be of negligible economic importance, resulting only in livers being discarded during meat inspection at slaughterhouses [11, 12]. In reality however, production performance losses in animals are often not associated with dicrocoeliosis, as the infection remains underestimated in field conditions because of its subclinical evolution [13]. The pathological effects related to dicrocoeliosis in ruminants can sometimes be overshadowed by concurrent liver infections (i.e. cystic echinococcosis, cysticercosis caused by *Taenia hydatigena*, fasciolosis); consequently, veterinarians and farmers may underestimate the importance of this disease [14–16]. Infected animals with a parasitic burden of under 1000 individuals of *D. dendriticum* usually do not show any clinical manifestations [17] and even infections with 4000 parasites can cause mild symptoms [8]. In fact, in previous work we reported that only 33.3% of practitioners diagnose dicrocoeliosis according to clinical symptoms [18].

In addition, sheep with *D. dendriticum* are often co-infected with other parasites (e.g. gastrointestinal and bronchopulmonary nematodes) making it quite difficult to identify the specific outcomes of each individual parasitosis [5].

Another aspect that may lead to an underestimation of dicrocoeliosis is that this parasitosis is generally not diagnosed with an appropriate coprodiagnostic analysis, thus infected animals are not identified [19]. As a consequence, the infection becomes increasingly persistent, with cumulative effects [19, 20]. Moreover, serological techniques do not provide reliable information for diagnosing dicroceliosis, although these tests may be useful when investigating prepatent infections [21].

Sardinia (Italy) has a long-established sheep farming tradition with over 3,200,000 sheep, which represent 45% of the entire stock of the Italian sheep population [22]. Due to the insularity and the high concentration of animals, which all belong to the Sardinian sheep breed, the island is regarded as a unique geographical area for epidemiological studies on parasites [15, 16, 23–28].

Most of the data on sheep dicrocoeliosis regarding Sardinia have demonstrated that this parasitosis is endemic, though much of the data is not recent [29, 30].

In this study, we investigated sheep dicrocoeliosis in Sardinia with particular emphasis on parasitological and molecular aspects in order to provide new insights into its epidemiology in extensive sheep farms.

## Methods

### Copromicroscopical survey in sheep farms

The sample size of the farms studied was estimated considering 15,387 Sardinian sheep farms (National Data Bank of the Italian Ministry of Health; https://www.vetinfo.it) with an expected *Dicrocoelium* spp. prevalence of 15%, and confidence level of 95% (http://www.raosoft.com/samplesize.html).

**Fig. 1:**
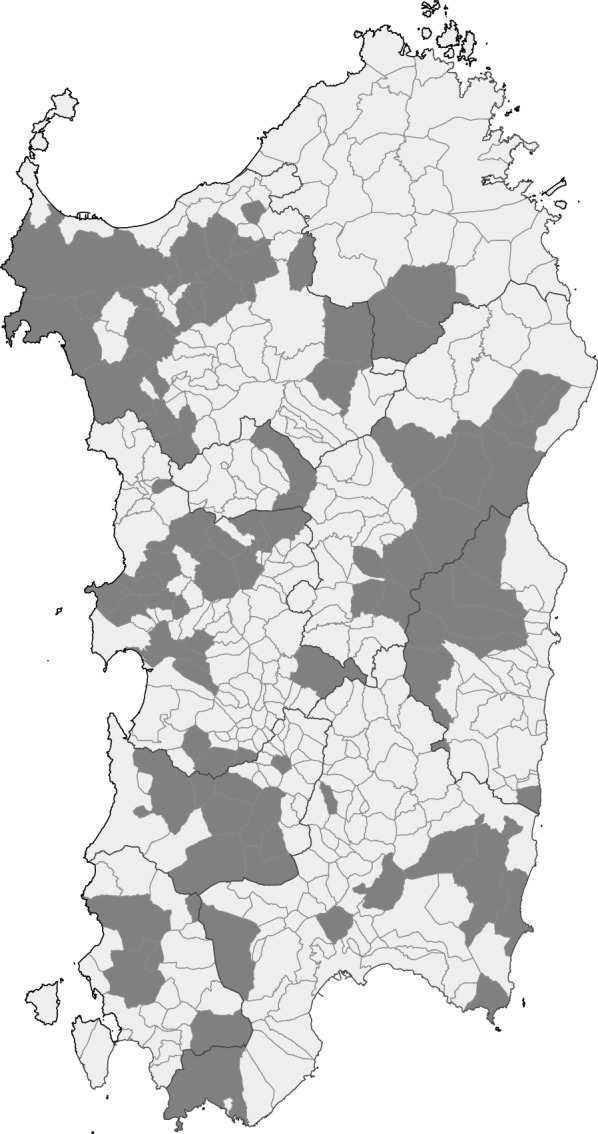
Map of the municipalities of Sardinia showing the sampling sites

A total of 190 dairy sheep farms in Sardinia (Fig. 1) were investigated from 2013 to 2014. Within each flock, 15 individual rectal faecal samples from sheep older than 3 years of age were collected. These samples were then split into three different faecal pools from five animals, which were then analysed using the FLOTAC® method with a heavy saturated zinc sulphate solution, specific gravity (SG) 1350) [31, 32].

Data were processed for each farm considering the eggs per gram (EPG) mean of the three faecal pools.

The data were then stratified by geolocalisation in the four provinces of Sardinia (Sassari, Cagliari, Nuoro and Oristano) (Fig. 1). Farms were grouped according to the EPG means values into four classes: (i) ≤ 50 EPG; (ii) > 50 and ≤ 100 EPG; (iii) > 100 and ≤ 300; (iv) > 300 EPG.

The mean intensity (MI) was obtained considering the arithmetic mean of the EPG values in the total number of the infected animals.

### Epidemiological survey in slaughterhouses

The sample size of the studied animals was determined considering a total of 3,206,821 heads of Sardinian dairy sheep (National Data Bank of the Italian Ministry of Health; https://www.vetinfo.it) with an expected *Dicrocoelium* spp. prevalence of 15%, and confidence level of 95% (http://www.raosoft.com/samplesize.html).

From 2013 to 2014, 770 Sarda sheep slaughtered in six different abattoirs in Sardinia, were submitted to anatomo-pathological examination to detect the liver parasites and to evaluate the typical thickened terminal bile duct (TTBD) lesions [33, 34], following the EEC Inspection Regulation No. 854 of 2004 (Annex 1, Section IV, Chapter II, point 5) [35] and the methods illustrated by Marcato [36].

To perform the anatomo-pathological examination, seven hepatic areas were selected: right lobe (RL) and left lobe (LL) of both the diaphragmatic face (DF) and visceral face (VF), quadrate lobe (QL), caudate lobe (CL) and finally the cut surface. For each area the severity/extension of the lesions indicative of a TTBD pattern were scored as follows: (0) absence of TTBD; (1) presence of rare TTBD; (2) ≤ 5 cm^2^; (3) 5–7.5 cm^2^; (4) 7.5–10 cm^2^; and (5) > 10 cm^2^.

Subsequently, according to the guidelines of the World Association for the Advancement of Veterinary Parasitology (WAAVP) [37], an incision on the gall-bladder wall was performed, and the entire liver parenchyma was cut into 0.5–1.0 cm slices, in order to identify and count the adult parasites. The parasitic burdens were classified into five classes, based on the number of parasites found in the organ: (i) ≤ 50; (ii) 50–100; (iii) 100–300; (iv) 300–1000; and (5) > 1000. Five adult *Dicrocoelium* spp. were taken from each liver in order to confirm the species based on published morphological keys [6].

### Genetic analysis

DNA from 15 adult *Dicrocoelium* spp. representing all four provinces of Sardinia was extracted using a commercial kit, PureLink® Genomic DNA Mini Kit (Invitrogen, Carlsbad, CA, USA) by following the manufacturer’s instructions. DNA samples were amplified by PCR for the regions internal transcribed spacer (ITS2) with the primers and the methods described elsewhere [6, 40]. PCR products were purified using a commercial kit (Nucleospin Gel and PCR Clean Up; Macherey-Nagel, Düren, Germany) and sent to an external sequencing service (Eurofins Genomics, Ebersberg, Germany). Sequences were assembled manually with the aid of the CLUSTAL W multiple alignment program [38], and analyzed using the basic local alignment search tool (BLAST) available on the NCBI website (https://blast.ncbi.nlm.nih.gov/Blast.cgi.).

### Statistical analysis

Data were processed using MINITAB v.12.1 (Minitab Inc., State College, PA, USA) and EpiInfo v.6.04 (CDC, Atlanta, GA, USA). A Chi-square test was performed to compare the prevalence in the four provinces. In order to compare the prevalence rates found in the different seasons, a chi-square trend test was used and odds ratio (OR) values were calculated. Mann–Whitney and Kruskal–Wallis non-parametric tests were used to compare the EPG means. Pearsonʼs correlation test was performed in order to evaluate the correlations between the parasite burden and TTBD score.

## Results

### Copromicroscopic survey

Ninety-seven out of the 190 examined farms were coprologically positive for *Dicrocoelium* spp. (51.1%; 95% CI: 43.91–58.07%). Quantitative coprological analysis of *Dicrocoelium* spp. showed a EPG mean (± standard deviation, SD) of 31.2 ± 68.7 and a MI of 61.1 EPG. Interestingly, dicrocoeliosis prevalence was significantly lower in the summer (37.5%) compared to the winter (90.9%), when the odds ratio (OR) values were four times higher than in other seasons (Table 1).

**Table 1:** Seasonal trend of prevalence, EPG mean excretion and odds ratio values for *D. dendriticum* in farm faecal pools samples

Season	Total no. of farms	No. of positive farms	% positive farms^a^	EPG Mean ± SD^b^	Odds ratio
Spring	13	9	69.2	26.8 (29.4)	1.00
Summer	120	45	37.5	22.1 (65.4)	0.27
Autumn	46	33	71.7	52.8 (80.3)	1.13
Winter	11	10	90.9	44.9 (68.8)	4.44

^a^*χ*^2^ trend = 11.558, *df* = 3, *P* < 0.0007

^b^Kruskal–Wallis test: *H* = 24.74, *P* < 0.0001

Table 2 shows the prevalence, farm EPG mean, MI values for *Dicrocoelium* spp. in the faecal pools and OR values in the four provinces. Regarding the *Dicrocoelium* spp. prevalence, the four provinces showed significant differences (*χ*^2^ = 23.89, *df* = 3, *P* < 0.0001); there were also statistically significant differences in EPG means (Kruskal–Wallis H-test: *χ*^2^ = 30.88, *P* < 0.0001). The province of Nuoro showed the highest prevalence and EPG means for dicrocoeliosis, as well as the highest OR values (OR = 9) (Table 2).

**Table 2:** Prevalence, EPG mean values, mean intensity and odds ratio values for *D. dendriticum* in farm faecal samples in each province of Sardinia

Province	Total no. of farms	No. of positive farms	Prevalence (%)^a^	EPG Mean^b^	Mean intensity (EPG)	Odds ratio
Cagliari	32	16	50.0	55.5	111.0	1.00
Oristano	44	22	50.0	21.2	42.4	1.00
Sassari	84	32	38.1	16.4	43.0	0.62
Nuoro	30	27	93.3	61.4	66.8	9.00

^a^*χ*^2^ = 23.89, *df* = 3, *P* < 0.0001

^b^Kruskal–Wallis test: *H* = 30.88, *P* < 0.0001

On-farm overall prevalence of *Dicrocoelium* by year was 36.3% (45/124) in 2013 and 78.8% (52/66) in 2014. There were statistically significant differences in prevalence between the two years (*χ*^2^ = 31.13, *df* = 1, *P* < 0.0001), as well as in the EPG means, which were 20.7 ± 63 EPG in 2013 and 50.8 ± 89.3 EPG in 2014 (Mann–Whitney U-test: *U* = 10554.0, *P* = 0.0004).

A total of 156 (82.1%) of the farms investigated were negative or with EPG mean values of ≤ 50 EPG, while 17 (9%) had EPG mean values of 50–100 EPG, 13 (6.8%) had EPG mean values of 100–300 EPG and only four (2.1%) had EPG mean values of > 300 EPG. These values were statistically different (*χ*^2^ = 443.09, *df* = 3, *P* < 0.0001).

### Epidemiological survey in slaughterhouses

The anatomo-histopathological examination of the livers showed a *Dicrocoelium* spp. prevalence of 25.5% (95% CI: 0.22–0.28%) (196/770), 54.1% of which harboured less than 50 adult parasites per organ, while only 3% harboured over 1000 parasites (Table 3).

**Table 3:** Prevalence and odds ratios of *D. dendriticum* in livers examined at abattoirs

Infection class^a^	No. of positive livers	Prevalence (%)^b^	Odds ratio exposure score
≤ 50	106	54.1	1.00
> 50 to ≤ 100	37	18.9	0.20
> 100 to ≤ 300	25	12.8	0.12
> 300 to ≤ 1000	22	11.2	0.11
> 1000	6	3.0	0.03

^a^No. of adult parasites

^b^*χ*^2^ trend =147.25, *df* = 3, *P* < 0.0001

**Fig. 2:**
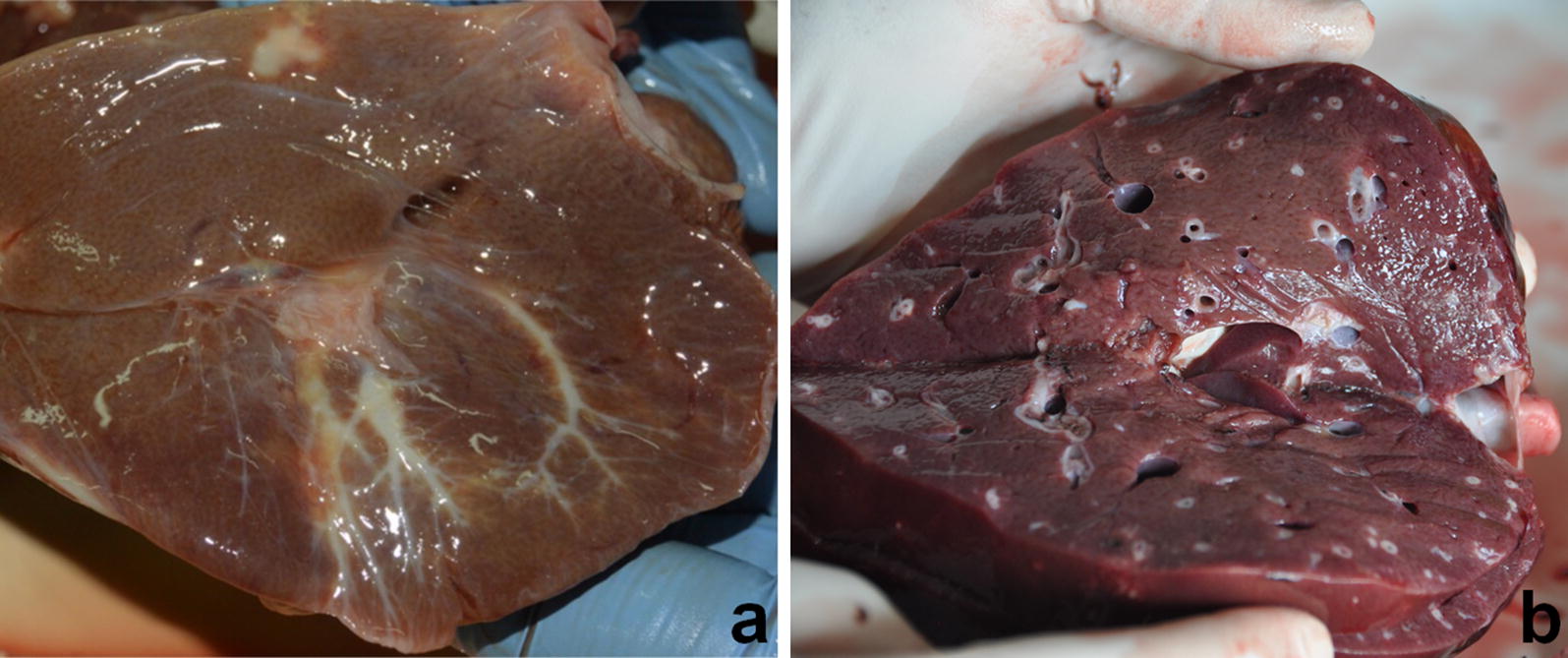
Thickened terminal bile duct (TTBD) on the surface (**a**) and cut surface (**b**) of the liver

TTBD on the surface and the cut surface were reported in 40.1% (309/770) and 15.3% (118/770) of examined livers, respectively (Fig. 2). The hepatic areas most involved were the RL of VF and CL with a prevalence of 24.8% (191/770) and 16.8% (129/770), respectively. TTBD was not observed in the quadrate lobe. Our results did not show any match between the presence of parasites in the examined livers (25.5%) and the TTBD both on the surface (40.1%), and the cut surface (15.3%) (*χ*^2^ = 121.62, *df* = 2, *P* < 0.0001). The score values were higher in RL VF than the other hepatic localizations. Detailed data are reported in Table 4.

**Table 4:** Score of livers reporting thickened terminal bile duct (TTBD) in the different examined areas: right lobe (RL) and left lobe (LL) of diaphragmatic face (DF) and visceral face (VF), caudate lobe (CL) and cut surface

Localisation TTBD	No. positive (%)	Score, no. positive (%)				
		1	2	3	4	5
RL DF	45 (5.8)	34 (4.4)	11 (1.4)	0 (0)	0 (0)	0 (0)
LL DF	5 (0.7)	5 (0.7)	0 (0)	0 (0)	0 (0)	0 (0)
RL VF	191 (24.8)	95 (12.3)	79 (10.3)	6 (0.8)	11 (1.4)	0 (0)
LL VF	124 (16.1)	67 (8.7)	51 (6.6)	6 (0.8)	0 (0)	0 (0)
CL	129 (16.8)	6 (0.8)	107 (13.9)	10 (1.3)	6 (0.8)	0 (0)
Cut surface	118 (15.3)	45 (5.8)	45 (5.8)	11 (1.4)	17 (2.2)	0 (0)

There was a significant positive correlation between the parasite burden and the scores for the severity/extension of TTBD as follows: RL of DF (*r* = 0.538, *P* < 0.0001), RL VF (*r* = 0.484, *P* < 0.0001), LL VF (*r* = 0.374, *P* < 0.0001), CL (*r* = 0.351, *P* < 0.0001) and the cut surface (*r* = 0.338, *P* < 0.0001). According to Colton [39], the correlation, based on reported *r*-values, between the parasitic burden and RL VF was moderate to good, while the correlations between the parasitic burden and the other hepatic localizations were quite good. Using the morphological examination, all *Dicrocoelium* spp. were identified as *D. dendriticum* [6].

### Genetic analysis

No intraspecific variations were observed for the ITS2 gene sequence (GenBank: MG004688) among the *D. dendriticum* isolates. In addition, the same isolates showed a homology of 99%, with the Iranian ITS-B haplotype isolate (GenBank: JQ966973) [40], and a homology of 99% and 98% with the Italian isolates DQ379986.2 [41] and EF547132.1 [6], respectively. The sequence alignment of ITS showed a T/A substitution at the 153 codon with an index diversity of 0.002, compared with the sequence of the above mentioned Iranian isolate.

## Discussion

The present survey provides an update of various epidemiological aspects of sheep dicrocoeliosis in Sardinia. Our copromicroscopic survey on the farms revealed the widespread presence of *D. denditricum*. However, the distribution of this parasite does not appear to be homogeneous across the island, with significantly more farms affected in the province of Nuoro, which is located in the central part of the island. It should also be highlighted that this area is characterised by the highest altitude with an average altitude of 496 meters) (http://www.comuni-italiani.it/20/clima.html) and the lowest average temperature compared with the other provinces (http://www.sar.sardegna.it/pubblicazioni/riepiloghimensili/mensili.asp).

The EPG means and OR values appear to be statistically higher in the winter in other regions of Italy [33] and also in other countries [42–44]. In Spain it has been reported that the mountainous pastures located over 600 meters and with temperatures of < 11.8 °C present the highest risk of infection with *D. dendriticum* [44]. These findings suggest that in some geographical areas and, especially during the winter, it is important to monitor and carry out anthelmintic treatments against *Dicrocoelium* spp. in sheep. We also found a different prevalence and EPG means values between the two years studied, thus suggesting that the epidemiology of the dicrocoeliosis could also be influenced by annual climatic conditions.

Our results show that the prevalence of dicrocoeliosis in Sardinia appears to be lower compared with other sheep-farming areas of Italy, such as Umbria (80%) [45], southern Apennines (67.5%) [1], Campania (67%) [46] and Basilicata (62%) [47].

Our survey demonstrated that inspections at slaughterhouses can detect the presence of the typical lesions due to *D. dendriticum* in the liver, and can thus be used to monitor the presence of chronic infections in a given flock. Underestimating the numbers of infected sheep is thus leading to the spread of parasitosis in Sardinia, which probably explains the high prevalence among sheep flocks on the island.

According to Ambrosi [33], infections with threshold values of under 100 adult parasites are not easily detected by copromicroscopical analysis. The same author [33] reported that approximately 7% of farms with EPG means values over 100 EPG could incur production losses. However, we found that only 3% of the examined livers in slaughterhouses showed a burden of over 1000 *D. dendriticum*. At the same time, the mild clinical signs might contribute to chronic infection and potentially to a loss of productive performance, which could be an interesting research line for further studies on this parasite.

Although previous papers have reported a high variability within *D. dendriticum* [48], both in terms of genetic and morphological parameters, no intraspecific variation was observed within our isolates and our results were consistent with findings in other surveys carried out in Italy [6, 41] and in Iran [40].

## Conclusions

This present study show the widespread presence of *D. dendriticum* in Sardinia and highlights the key role of abattoirs and of the coprological analysis in the monitoring of parasitic diseases, through which farmers and practitioners can be given the data needed for diagnosing *D. dendriticum* and thus for setting up specific anthelmintic treatments.

## Data Availability

All relevant data are included in the article. The newly generated sequence was submitted to the GenBank database under the Accession Number MG004688.

## Abbreviations

TTBD: thickened terminal bile duct
EPG: eggs per gram
MI: mean intensity
OR: odds ratio
RL: right lobe
VF: visceral face
CL: caudate lobe
QL: quadrate lobe
DF: diaphragmatic face
LL: left lobe
ITS: internal transcribed spacer
NDB: National Data Bank
SD: standard deviation
